# Editorial: Artificial intelligence in finance and industry: Highlights from 6 European COST conferences

**DOI:** 10.3389/frai.2022.1007074

**Published:** 2022-09-09

**Authors:** Andreas Henrici, Jörg Osterrieder

**Affiliations:** ^1^School of Engineering, ZHAW Zurich University of Applied Sciences, Winterthur, Switzerland; ^2^Department of High-Tech Business and Entrepreneurship, University of Twente, Enschede, Netherlands; ^3^Bern Business School, Bern University of Applied Sciences, Bern, Switzerland

**Keywords:** artificial intelligence, Fintech, machine learning, explainability of AI, decision making

This Research Topic contains contributions from six research conferences on Artificial Intelligence (AI) in Finance and Industry. The conferences have been organized since 2016 at Zurich University of Applied Sciences in Switzerland^1^ and have attracted a large audience of participants from industry and academia. Our conference series is motivated by the idea that the possibilities and challenges of AI applications in real-world contexts can be understood only by a bottom-up approach, i.e., by considering as many case studies as possible from as many different economic sectors as possible. Moreover, given the fact that almost all economic sectors now claim themselves to be technology-based, it is an urgent necessity to gain an overview of the specific challenges encountered in the practical implementation of abstract AI ideas.

Artificial Intelligence in Finance and Industry has gained substantial interest both in industry and academia. This conference series can be seen as a continuation and extension of various Research Topics in artificial intelligence.

Giudici et al. (2019) show the connection between Financial Technology and AI, in the context of a multi-year European research programme.

The topic collection contains three papers on AI applications in the financial sector and one paper discussing the degree of human control on AI systems in general, mainly from an ethical perspective. This emphasizes the need for simultaneous progress on both the technical and the ethical side of AI applications, if we want these applications to be successful and have a high level of trustworthiness outside the inner circle of technology specialists.

In the article “*A Matrix-Variate t Model for Networks*” by Billio et al. network analysis is used as a tool to study relationships between financial firms. In addition, a new statistical tool is proposed for the pre-processing or de-noising of the network data before the proper network analysis. The background of their work is the extraction of unobserved networks from financial time-series data, which, however, is contaminated with various types of errors. These inconsistencies call for statistical tools to avoid conclusions based on erroneous or imprecise assumptions and to create a certain robustness in the extraction of network structures. To this end, the article proposes a new Bayesian model for such network data based on data augmentation and special prior distributions that allow an efficient posterior sampling. The usefulness of the proposed method is then demonstrated in an application to de-noising network data from stock market returns.

In the article “*The Perils of Misspecified Priors and Optional Stopping in Multi-Armed Bandits*” by Loecher, the effect of optional stopping in a class of multi-armed bandit experiments is investigated. Whereas such bandits can, in principle, run indefinitely, by introducing intelligently chosen stopping conditions, one can make these principally indefinite experiments practically feasible. Moreover, instead of the usual test/train-framework from machine learning with a suitable cost function, in these artificially stopped bandit experiments, one uses the “terminal regret” as the difference between the optimal outcome and the one chosen by the stopping rule as a metric to assess the performance of the stopping procedure.

In the article “*Let Me Take Over: Variable Autonomy for Meaningful Human Control*” by Methnani et al. various aspects of the optimal degree of human control over AI systems are discussed, and the article, in particular, argues that the development of systems with a variable autonomy, respectively, human control is a suitable tool to satisfy the core ethical values of AI systems. The background of this article is the need for AI tools that are following the legal and ethical values of our society, in particular the desire for responsible AI systems. Specifically, they analyse how the core values of accountability, responsibility and transparency can be established in AI systems. In this sense, the article discusses various implications of “meaningful human control,” which refers to control frameworks in which humans are not only controlling critical decisions but also critical steps in building up the underlying AI systems. With the proposed variable autonomy of a system, the amount of human control is imagined to be dynamically adjustable to retain as much control over the system as possible without having to take an unmanageable amount of decisions at the operational level which would question the functionality and usefulness of the AI system under consideration.

Finally, in the article “*Review of Multi-Criteria Decision-Making Methods in Finance Using Explainable Artificial Intelligence*” by Cernevičiene and Kabašinskas, the need for explainability of AI-based decisions serves as a motivation for using multi-criteria decision-making (MCDM) particularly in financial decisions. Examples of MCDM are portfolio optimisation, pension fund evaluation or bankruptcy prediction. For an application of explainable AI in credit risk management, Misheva et al. (2021) implement two advanced *post-hoc* model agnostic explainability techniques called Local Interpretable Model Agnostic Explanations and Shapley Additive explanations to publicly available P2P lending datasets.

As one particular flavor of AI, the importance of neural networks has been realized and they are used in a variety of contexts.

Osterrieder et al. (2020) as well as Hirsa et al. (2021a) apply neural networks to the VIX Index and show how to both replicate and predict its values.

Hirsa et al. (2021b) have combined neural networks with reinforcement learning to create a portfolio of financial instruments from different asset classes and predict their prices.

As in the article by Billio et al. the data available as inputs for a decision-making process can be incomplete or noisy, which raises the question of the robustness of the decisions taken based on these incomplete or noisy data. The article then proceeds to give an overview over possible types of AI-based methods, such as Artificial Neural Networks, Rule-Based Models, Kernel Methods, Fuzzy Modeling of Metaheuristics models. As in the article by Methnani et al., the explainability issue is discussed and taken as one of the main criteria for the evaluation of the proposed methods; again, the need for explainability comes one the one hand from the need to establish public trust in the results of AI methods, and on the other hand from the need to comply with regulatory issues.

In various contexts, neural networks are used to generate synthetic data. For an overview of approaches in Finance, see Eckerli and Osterrieder (2021). Samuel et al. (2021) as well as Pfenninger et al. (2021) have applied Wasserstein GANs to financial time-series to both augment existing data as well as run simulations on those artificially generated time-series. In this sense, the article collection discusses technical and ethical issues arising in the implementation and operationalisation of AI systems. Although the applications discussed in the article series are mainly centered around financial topics, the issues and problems arising in these applications are by no means restricted to AI applications in finance. Quite to the contrary, if one views AI as a new paradigm which has to be used in some form by all technology-based industries, at least if they want to remain successful, these questions of optimizing AI algorithms and creating public trust in the results of these algorithms have to be tackled by all industries.

## Author contributions

All authors listed have made a substantial, direct, and intellectual contribution to the work and approved it for publication.

## Conflict of interest

The authors declare that the research was conducted in the absence of any commercial or financial relationships that could be construed as a potential conflict of interest.

## Publisher's note

All claims expressed in this article are solely those of the authors and do not necessarily represent those of their affiliated organizations, or those of the publisher, the editors and the reviewers. Any product that may be evaluated in this article, or claim that may be made by its manufacturer, is not guaranteed or endorsed by the publisher.

## Footnotes

^1^Head of the Organizing Committee: Prof. Dr. Jörg Osterrieder.
